# The highly pathogenic strain of porcine deltacoronavirus disrupts the intestinal barrier and causes diarrhea in newborn piglets

**DOI:** 10.1080/21505594.2024.2446742

**Published:** 2025-01-06

**Authors:** Xin Yao, Wei-Hong Lu, Wen-Ting Qiao, Yu-Qian Zhang, Bao-Ying Zhang, Hui-Xin Li, Jin-Long Li

**Affiliations:** a College of Veterinary Medicine, Northeast Agricultural University, Harbin, PR China; b State Key Laboratory for Animal Disease Control and Prevention, Harbin Veterinary Research Institute, The Chinese Academy of Agricultural Sciences, Harbin, PR China; c Key Laboratory of the Provincial Education Department of Heilongjiang for Common Animal Disease Prevention and Treatment, Northeast Agricultural University, Harbin, PR China; d Heilongjiang Key Laboratory for Laboratory Animals and Comparative Medicine, Northeast Agricultural University, Harbin, PR China

**Keywords:** Porcine deltacoronavirus, CH/LNFX/2022, evolutionary analysis, neutralizing epitope mutations, validation of regression

## Abstract

Porcine deltacoronavirus (PDCoV) is increasingly prevalent in newborn piglets with diarrhea. With the development of research on the virus and the feasibility of PDCoV cross-species transmission, the biosafety and the development of pig industry have been greatly affected. In this study, a PDCoV strain CH/LNFX/2022 was isolated from diarrheal newborn piglets at a farm in China. A genome-wide based phylogenetic analysis suggests that 97.5% to 99.2% homology existed in the whole genomes of other strains. Five amino acid mutations are seen for the first time in the S protein. By constructing 3D models, it was found that the S1-NTD/CTD and S2-HR-C regions produced structural alterations. Protein functional analysis showed that the structural changes of the three regions changed the epitope of S protein, the O-GalNAc glycosylation site and the 3C-like protease cleavage site. In addition, oral administration of 10^7^ TCID_50_ CH/LNFX/2022 to newborn piglets successfully reproduced obvious clinical signs of piglets, such as diarrhea and dehydration. Meanwhile, PDCoV antigen was detected by immunofluorescence in the small intestine, and microscopic lesions and intestinal mucosal barrier destruction were detected by histological observation and scanning electron microscopy. Our study confirmed that porcine coronavirus strains increased pathogenicity through evolution, damaged the intestinal barrier of newborn piglets, and caused diarrhea in pigs. This study provided the candidate strains and theoretical basis for establishing the prevention and control system of vaccine and diagnostic methods for piglet diarrhea.

## Introduction

Porcine deltacoronavirus (PDCoV) was first reported in 2012 in Hong Kong, China [1]. Until now, it has been widely prevalent in American regions such as the United States, Mexico, Canada and in Asia such as China, Japan, Korea, Thailand [2–5]. As a pathogen causing acute diarrhea and vomiting in pigs, it has caused significant economic losses to the global swine industry since its discovery [4,6,7]. PDCoV, which has a positive sense single stranded RNA genome of 25.4 kb in length, is a member of the genus Deltacoronavirus in the Coronaviridae family [8,9]. The genome, in turn, consists of: 5’UTR, open reading frame 1a/b (ORF1a/b), spike (S), envelopments (E), membrane (M), nonstructural protein 6 (NS6), nucleocapsid (N), nonstructural protein 7 (NS7), and 3’UTR [10].

The S protein is the most variable protein in PDCoV, with only 96.0%–100% amino acid sequence divergence between the American and Asian strains [11]. The S protein is placed on the envelope surface and plays an important role in viral recognition and adsorption to host cells [12]. The S protein of PDCoV includes two regions, S1 and S2, with the S1 domain including the NTD and CTD, containing neutralizing antibody epitopes, where the RBD located at the CTD is responsible for receptor recognition [13,14]. S2 is relatively conserved and mediates membrane fusion, including CH-N, FP, HR-N, CH-C, and HR-C [15].

PDCoV is infectious to pigs of different ages, especially newborn piglets with the same clinical manifestations as swine epidemic diarrhea virus and swine infectious gastroenteritis virus [16,17]. The co-infection of PDCoV and PEDV cannot be ignored, the proportion of PDCoV and PEDV co-infection has increased in recent years compared with other common piglet epidemics, and studies have shown that this is more severe and difficult to treat than infection alone [18]. The latest studies show that PDCoV infects calves and poultry while utilizing APN as a receptor to enter human cells, implying that the transmissibility of PDCoV makes it a potential high-risk novel coronavirus pathogen and that vaccine development and diagnostic methods for its establishment have important public health implications [19–22].

In this study, the highly pathogenic PDCoV strain CH/LNFX/2022 showed changes in the protein helical structure at S1-NTD, S2-HR-C, and S1/2 junctions compared with other epidemic strains, and changed the epitopes of existing epidemic strains, which affected the progress of 6-helix bundle (6HB) and viral membrane fusion. In addition, regression experiments confirmed that diarrhea in newborn piglets was caused by disruption of the intestinal mucosal barrier by highly pathogenic CH/LNFX/2022. Together, these results further complement the spread and evolution of PDCoV and provide new insights for vaccine development and diagnostic approaches.

## Materials and methods

### Collection of specimens and identification of pathogens

Piglets were collected from a diarrhoeal pig farm of Liaoning Province, China, in 2022 with clinical symptoms of vomiting, watery diarrhoea and death from dehydration. Nucleic acid extraction and reverse transcription were performed using Trizol™ (15596018CN, Thermo) and PrimeScript™ RT Master Mix (RR036Q Takara) on collected small intestinal tissues from diarrhea pigs. The real-time PCR method was consistent with previous work [23]. The N gene plasmid was used as a positive control to calibrate viral copy number and was kept by the laboratory.

### Cell culture and diarrhea virus adaptive isolation

Ten percent fetal bovine serum (SH30088.03, Hyclone) was added in Dulbecco’s modified eagle’s medium (DMEM, SH30081.01, Hyclone) for in vitro experiments. Small intestinal lesions with positive PCR results were ground and mixed 1 g: 10 mL using DMEM containing 100 U/mL penicillin streptomycin (SV30010, Hyclone). The filtered liquid was labeled with P0 (0.22 μm, BS-PES-22, Biosharp), and Swine Testis (ST) cells cultured to 80% confluence in T75 cell flasks (708003, NEST) were infected as adsorbate. After incubation at 37°C with 5% CO_2_ for 72 h, freeze–thaw was repeated at low temperature. The freeze–thaw solution was collected, and the filtered P1 passage virus labeled solution was used to observe the cytopathic effect and virulence study (*n* = 3) after P10.

### Construction of recombinant plasmids for N

Specific N gene primers (Comate Bioscience, China) were designed based on the published sequence of CH/LNFX/2022 (ON968724.1). N-F/R PCR was used and the PCR products were recovered (DP219, TIANGEN) and connected to T-Vector pMD19 (Simple, 3271, Takara) according to manufacturer’s as well as plasmids from individual colony cultures. Sanger sequencing was performed after extraction (Comate Bioscience, China), and the sequencing results were consistent with the database (Supplementary Fig. S1). The resulting plasmids were diluted 10^2^ to 10^7^ times, and a standard curve was established by real-time PCR, which was used as a reference for subsequent absolute quantification of virus replication.

### Detection of TCID_50_ after virus passaging

ST cells (2 × 10^6^/mL) were infected with PDCoV, subjected to 10 serial dilutions of PDCoV, and incubated for 2 h at 37°C in 5% CO_2_. After washing twice with sterile PBS, DMEM plus 2% fetal bovine serum was added to the cell plates (200 μL per well) and incubated at 37°C with 5% CO_2_ for 24 h before TCID_50_ was determined using the Reed–Muench method (*n* = 8).

### Indirect immunofluorescence assay (IFA)

ST cells cultured to 80% confluence in 6-well plates (3736, Corning) were infected with PDCoV CH/LNFX/2022 for 24 h. The cells were fixed with 4% paraformaldehyde for 30 min, perforated with 0.2% TritonX-100 (P0096, Beyotime) after three washes with PBS for 10 min, washed again three times with PBS, and incubated at BSA (SRE0096, Sigma) 37°C for 20 min. After washing, anti-PDCoV monoclonal antibody incubation was used (SD-4-5, Medgene Labs). After washing three times and added Alexa fluor 488 (A0428, Beyotime) conjugated goat anti-mouse IgG. After being protected from light treatment, cells were washed and observed (Leica, Germany). Tissue sections were subjected to color development in the same manner, except for incubation with Alexa fluor 594 conjugated goat anti-mouse IgG (A-11005, Thermo) (*n* = 3).

### whole genome sequencing of pathogenic samples

Library preparation and sequencing at the MAGIGENE Technology Co., LTD (MAGIGENE, China).

### Sequence analysis

The respective protein amino acid sequences of CH/LNFX/2022 and the reference strains (Supplementary Table 1) were aligned using DNAMAN (v6.0). MEGA6 software was used to build phylogenetic trees genome-wide and for each gene, and the neighbor joining (NJ) method set the guide value to 1000 repeats. ITOL has been involved in the esthetic process of strain phylogenetic trees. The MegAlign (v7.1.0.44) program was used to analyze the nucleotide homology of the genome and individual genes of the reference strain and CH/LNFX/2022, and the results were used for analysis by Origin generation Heatmap (v2021).

### Protein 3D structure model and function prediction

Individual protein tertiary structures were homology modeled by Phyre2 and SWISS-MODEL. DNAMAN alignment results predicted amino acid helix trends and FirstGlance in JMOL verified the position of amino acid mutations. Using NetCorona-1.0 to predict the location of coronavirus 3C-like protease cleavage proteases in proteins, and using NetNGlyc-1.0 to predict O-GalNAc (mucin type) glycosylation sites in mammalian proteins. Transmembrane functional changes of S protein from CH/LNFX/2022 were predicted by TMHMM (v2.0).

### Animal experiments

The experiment was carried out after the newborn piglets were fed adaptively with artificial milk replacement for 48 h. In vivo infection of PDCoV (*n* = 10) was described in animal experiments according to the National Guidelines for the management and use of Laboratory Animals (CNAS-CL06: 2018) and ARRIVE (Animal Research: Reporting of In Vivo Experiments), and approved by the Animal Experiment Ethics Committee of Northeast Agricultural University (NEAUEC20230383). Fourteen 1-day-old male newborn Large White pigs weighing 1.2 ± 0.1 kg from Qinggang farm were used in the experiment. The experimental animals were in field condition with their mothers, healthy and unvaccinated. Animals were randomly divided into infection (H.P.I) groups (*n* = 10) and control (Mock) groups (*n* = 4) using a computer-based random order generator at the Laboratory Animal Center of Northeast Agricultural University. The two groups of animals were assigned to two separate rooms with the same experimental conditions, and the piglets were raised freely in pens. Artificial milk substitutes were fed every 4 h for 48 h of adaptation. The 1 mL of PDCoV mixed milk (TCID_50_ ≈ 10^7^) was orally infected on the third day, and the same volume of sterile PBS buffer was used to mock infection of newborn pigs. Each piglet was treated as an experimental unit and included in the study as a Mock group uninfected or an H.P.I group infected. Otherwise, they are excluded. All 14 experimental units in this study met the criteria and were included in the study. In addition, the investigators who performed randomization (the fourth author) and the investigators who analyzed the data (the second author) were unaware of the group assignment, but the rearing investigators (the first and fifth authors) were aware of the group assignments due to the distinct clinical features.

### Histological analysis

After the jejunum and colon were isolated and fixed in tissue fixative solution for more than 24 h, they were dehydrated, embedded, and sectioned. Hematoxylin and eosin were used for staining, and H&E staining of the colon was performed after deparaffinization. IFA analysis of jejunum was performed using specific PDCoV N protein antibody staining (*n* = 3).

### Electron microscopy

PDCoV through 3000 × g differential centrifugation 10 min, then in 4°C, 30 min 12,000 × g differential centrifugation for clarification. After passing through a 0.22 μm filter, viral particles in the supernatant were centrifuged at 140,000 × g for 3 h and then re-suspended in 500 μL phosphate buffer (PBS) and overlaid on a discontinuous (15% −40%) sucrose density gradient. After ultracentrugation at 140,000 × g for 2 h, a 2 mL fraction was collected, and sucrose was removed by additional ultracentrugation. Virus particles were dispersed in 100 μL PBS and negative stained with 2% phosphotungstic acid (pH 7.0) for transmission electron microscopy (TEM) (*n* = 3). After the jejunum was isolated and fixed at 4°C in glutaraldehyde for more than 1.5 h, it was rinsed two to three times with PBS. Dehydration was performed using sequentially 50%, 70%, 90%, and 100% ethanol, with 100% ethanol twice for 10 min each. Replacement was performed using 50% and 100% tert-butyl alcohol sequentially, with 100% tert-butyl alcohol twice for 15 min each. The samples were dried at −20°C and glued to the sample stage. After coating, scanning electron microscopy (SEM) observation was performed (*n* = 3).

### Analysis of recombination

Use RDP included seven procedures, such as Chimaera BootScan, 3seq, GENECONV, MaxChi, and SiScan. This study shows that the recombinant results hold in all seven procedures (*p* <0.05).

### Statistical analysis

The software GraphPad Prism (version 8.3) was used for statistical comparison, where the Student's t-test was used to analyze the data (*, *p* < 0.05; **, *p* < 0.01; ***, *p* < 0.001). Fluorescence imaging was quantitatively analyzed using ImageJ (version 1.8), and a difference coefficient *P* less than or equal to 0.5 was considered statistically significant in the Heatmap. Data that were not significant or not statistically significant were not labeled.

## Results

### Isolation and cell adaptation culture of CH/LNFX/2022

**Figure 1.**
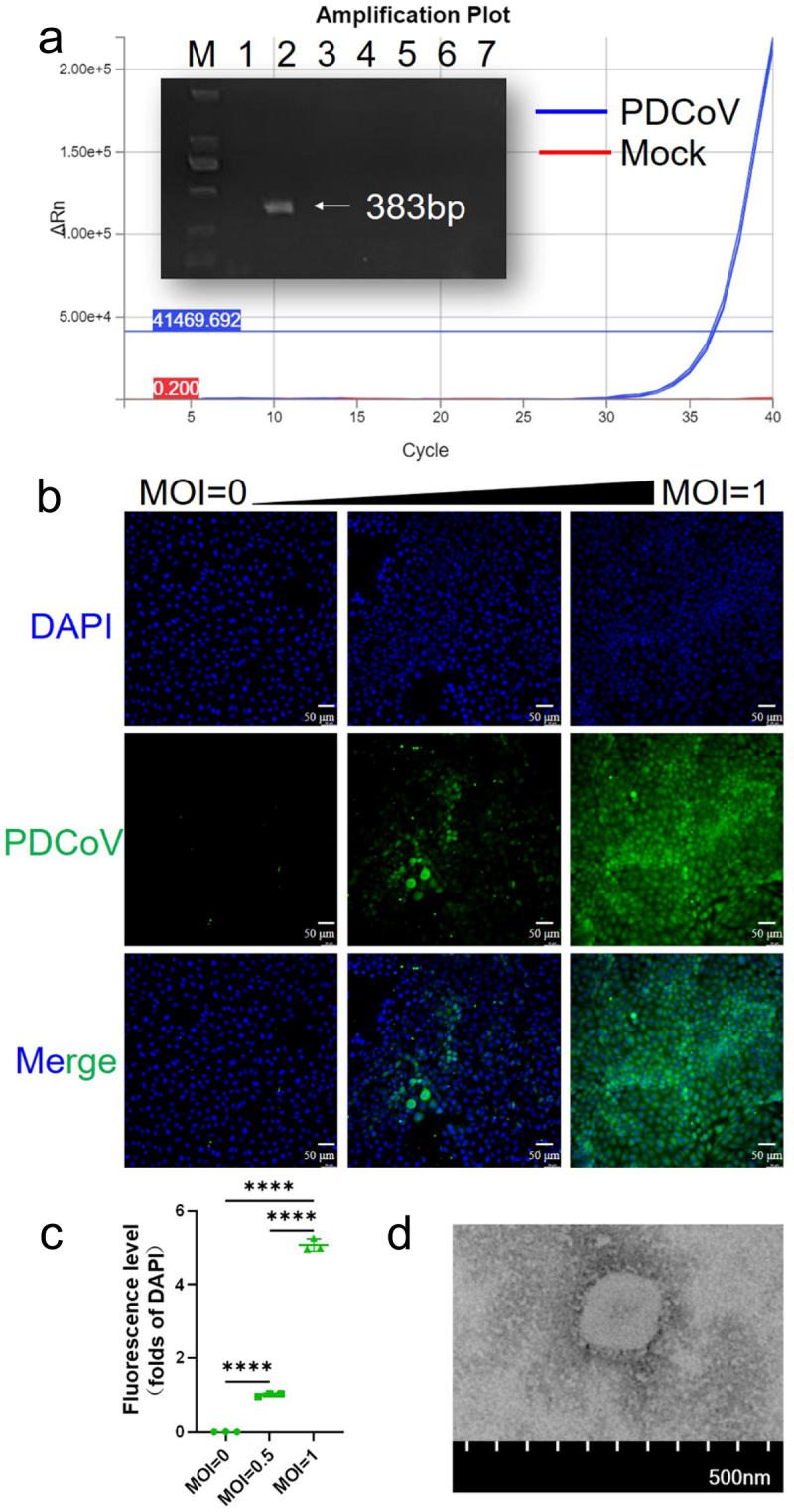
PDCoV isolation and culture. (a) Assays for PCV2, PDCoV, TGEV, PRRSV, PRV, PEDV, BVDV were performed sequentially. (b) PDCoV P10 infected ST cells, and the MOI = 0, 0.5, 1, and 24 h later for immunofluorescence detection (*n* = 3). (c) PDCoV-N protein green fluorescence was quantified using the DAPI fluorescence value as a standard (*n* = 3, ****, *p* < 0.0001). (d) The supernatant of infected tenth passage lysates was collected for TEM (*n* = 3).

Diarrhea, dehydrated lethal piglets appeared in 2022 in Liaoning Province farm of China, and the RT-PCR and RT-qPCR results indicated that PDCoV was positive, while other pathogens with similar symptoms were negative (Figure 1a and Supplementary Table 2). The small intestine and its contents were homogenized and an adsorbate was made to infect ST cells, and the harvested viral fluids were labeled as P1 and serially passaged until P10 for subsequent studies. After 24 h of infection of ST cells at P10, IFA showed purposeful fluorescence using a monoclonal antibody against the PDCoV-N protein, which was absent in the negative control, and green fluorescence increased as the infection coefficient rose (Figure 1b). The fluorescence quantification results clearly showed the good adaptability (****, *p* < 0.0001) of PDCoV on ST cells (Figure 1c). Transmission electron microscope results showed that there were virus particles with the same size and shape, and there were crown-shaped protrusions outside the cell membrane (Figure 1d). These results confirmed the existence of PDCoV.

**Figure 2.**
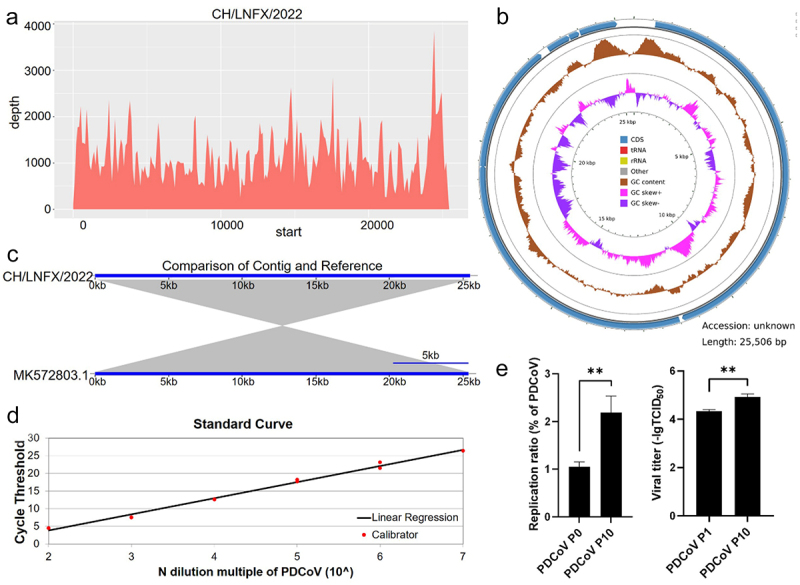
Sequencing and cellular adaptation of PDCoV (a) genome sequencing depth results. (b) Sequencing fragmentation assembly results. (c) Assembly results were aligned to the database. (d) N recombinant plasmids were prepared to generate a standard curve (*n* = 3). (e) Virus copy numbers of virus fluid from the adsorbent fluid of the disease and from the tenth generation of culture were compared (*n* = 3), and virus infectivity was compared between the first and tenth generation viruses (*n* = 8) (** *p* < 0.01).

Although we saw a single virion in the TEM field, we performed next-generation sequencing on P10 to ensure viral purity and for further analysis. The sequencing results showed that the sequencing depth was neat, and the full length of the fragment was 25,506 bp after splicing, which was consistent with the PDCoV genome length, by database alignment, and the sequencing strain was found to be the closest to SCNC201705 (MK572803.1) (Figure 2a, b and c). To better investigate viral replication, we constructed N-gene recombinant plasmids targeting conserved sequences of PDCoV and plotted a standard curve (Figure 2d). A comparative examination of the P1 and P10 generations of PDCoV revealed that the tenth generation of virions replicated at a higher rate than the first generation, and the cell infectivity titer was also higher than that of the first generation (Figure 2e).

### Whole genome sequence characterization of CH/LNFX/2022

**Figure 3.**
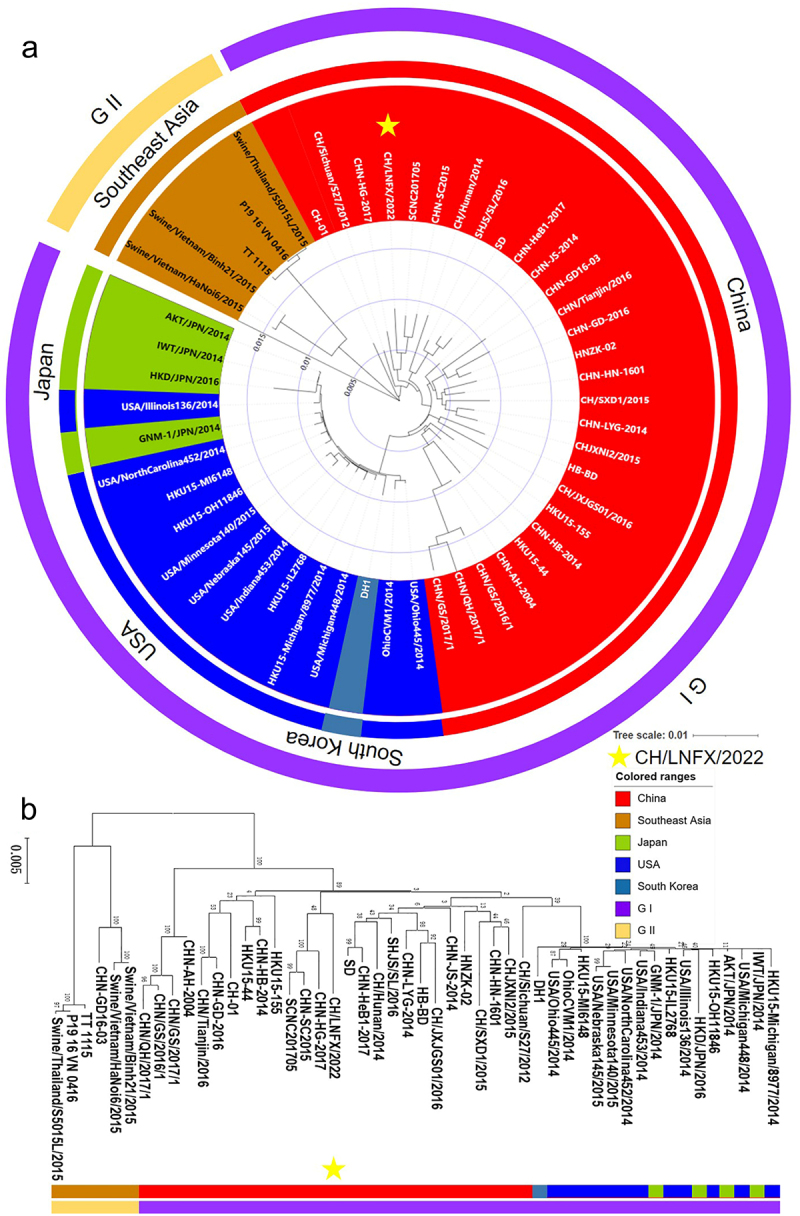
The whole genome and S protein sequences of the CH/LNFX/2022 strain were analyzed for genetic evolution. The legend of A and B is the same. (a) The CH/LNFX/2022 and 50 PDCoV strains were subjected to evolutionary analysis and divided into two subtypes, GI and GII, with three lineages of strains from China/America, Japan, and Korea/Southeast asia. (b) S protein is the same as above.

The isolate named CH/LNFX/2022 was submitted to GenBank under the accession number ON968724.1 after the complete genome sequence was deduced on the Illumina platform. A total of 25,506 nucleotides were detected for this strain, including ORF1a (nt 572–11441), ORF1b (nt 11,441–19368), S (nt 19,350–22829), E (nt 22,823–23074), M (nt 23,067–23720), NSP6 (nt 23,720–24004), N (nt 24,025–25053) and NSP7 (nt 24,119–24721). Full gene phylogenetic analysis showed that PDCoV is currently mainly concentrated in the Americas and endemic in Asia, but the American strains are more closely related to Asian strains and belong to the GI subtype, whereas the endemic strains in Southeast Asia are more distantly related and belong to the GII subtype (Figure 3a). The isolate CH/LNFX/2022 marked by yellow Pentagram belonged to the GI subtype and was close to other isolates in China, and the results of further S gene evolution analysis were consistent with it (Figure 3b). The whole genome of CH/LNFX/2022 shared 97.5%–99.2% identity with other strains and was most similar to SCNC201705 by analysis of PDCoV homology, while Southeast Asian strains showed 97.5% nucleotide homology (Supplementary Fig. S2). The genomic homology normalized heatmap showed significant differences compared to the genomes of the other strains, and this result was repeated for the S gene (Supplementary Fig. S3).

### The 5-aa mutation alters the spatial conformation of the S protein of PDCoV

**Figure 4.**
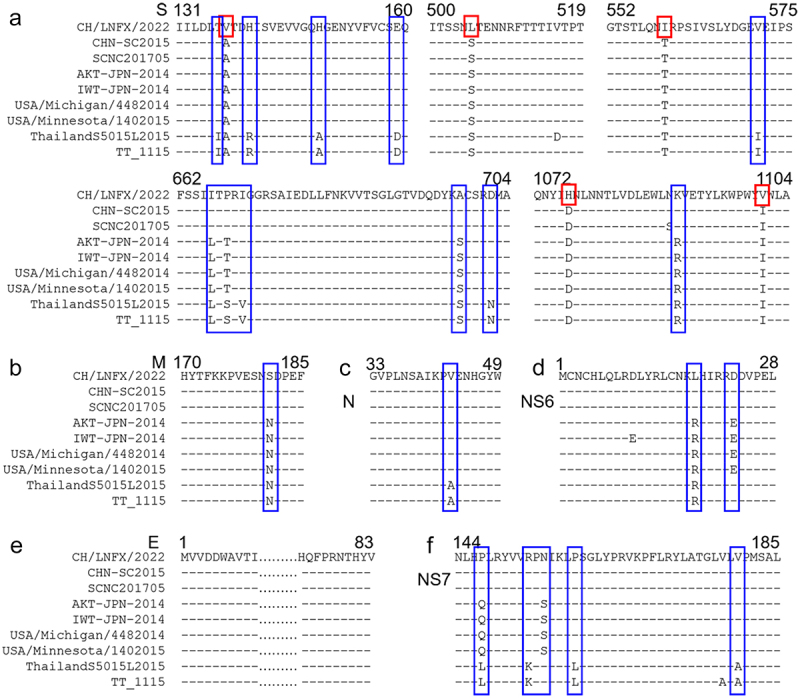
Two strains per region were randomly picked for alignment. (a) S protein to contrast. (b) M protein to contrast. (c) N protein to contrast. (d) NS6 protein to contrast. (e) E protein to contrast. (f) NS7 protein to contrast. The red box represents the unique evolutionary characteristics of CH/HLJJS/2022, and the blue box represents the characteristics of each lineage.

Amino acid alignments and analyses of two randomly selected Chinese strains, Japanese strains, American strains, and Southeast Asian strains were performed using DNAMAN. It showed 5-aa mutations on the S protein, A – V (aa 137) with the NTD structure, D – H (aa 1076) and I – V (aa 1101) located in the HR-C domain, S-L (aa 505), and T-I (aa 559) at the S1/2 junction (Figure 4a). Interestingly, we found the presence of partial amino acid specific conservation except for the E protein within strains from various regions, and these symptoms were the basis for the appraisal of PDCoV subtypes. Examples are GI/GII group SEQ/SDQ (aa 158–160) and Chinese/American or Japanese/Southeast Asian strains ITPRI/LTTRI/LTSRV (aa 666–670) in the S protein, GI/GII group NSD/NND (aa 180–182) in the M protein, PVE/PAE (aa 42–44) in the N protein, Chinese strains/other regional strains KLH/KRH (aa 17–19) in the NS6 protein and Chinese/American or Japanese/Southeast Asian strains RPN/RPS/KPN (aa 153–155) in the NS7 protein (Figure 4).

**Figure 5.**
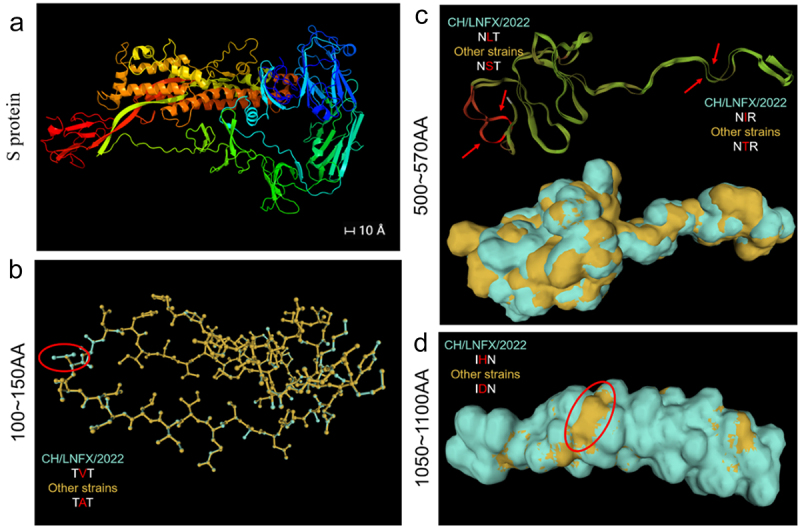
Detailed alignment of the S protein model to the reference strain. (a) S protein modeling results for CH/LNFX/2022. (b) The S protein 100-150AA range was modeled and the protein tertiary structure has a specific bulge at A – V (aa 137). (c) The S protein 500-570AA range was modeled and the protein tertiary structure has a specific bulge at S-L (aa 505) and T-I (aa 559). (d) The S protein 1050-1100AA range was modeled and the protein tertiary structure has a specific bulge at D – H (aa 1076).

Prediction of spatial helix trends of CH/LNFX/2022 and other strains’ proteins using DNAMAN. The A-V mutations located in the NTD domain shifted the strains down by COIL and up by STRAND (Supplementary Fig. S4A). S-L and T-I mutations located at the S1/2 junction raise the maximum likelihood HELIX and STRAND scores, respectively, while suppressing the second likelihood COIL (Supplementary Fig. S4B). D-H and I-V mutations located in the HR-C domain suppressed the maximum scored coil, facilitating the second scored (Supplementary Fig. S4C). To more intuitively embody the mutational impact, we quantified the predicted outcomes, embodied using line charts (Supplementary Fig. S4D). Modeling of the spatial structure of each protein from isolates and other strains showed in contrast no significant changes in the structure of the proteins except for the S protein (Figure 5a and Supplementary Fig. S5). To accurately identify a range of changes caused by the 5-aa mutation, we focused on the simulation scope of the mutation to identify potential changes at the mutation site. It was shown that 5-aa in the S protein altered the tertiary structure of the NTD, S1/2, and HR-C domains, with deflection of two helices of S1/2 by S-L and T-I, NTD, and HR-C protein protrusions by D-H and A-V (Figure 5b, c and d).

### 5-aa altered S protein antigenic epitopes and function

**Figure 6.**
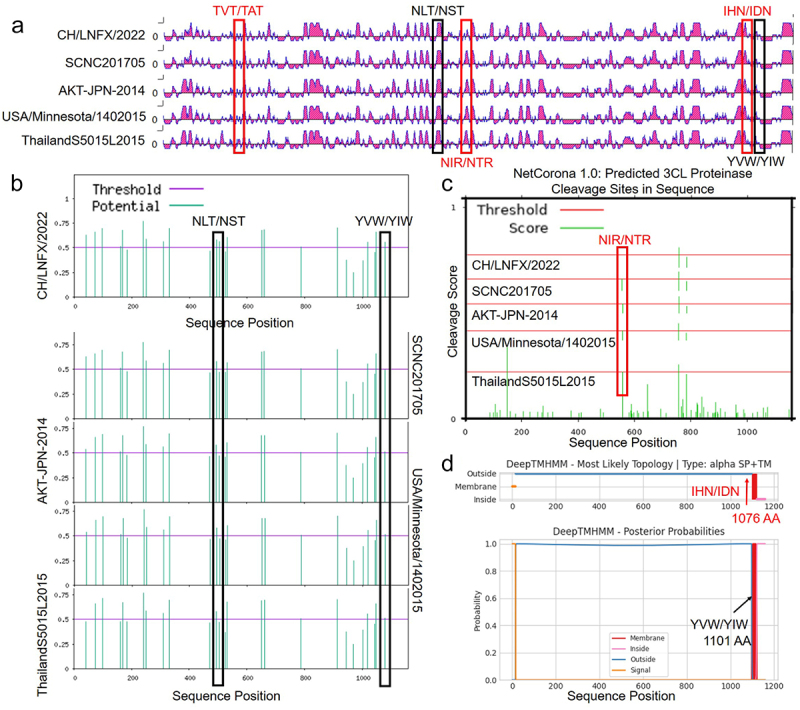
Protein function prediction. (a) Primary epitope comparative analyses of five regionally representative strains and CH/LNFX/2022 strain revealed 3-aa mutations out finding specific changes. (b) O-GalNAc (mucin type) glycosylation sites in mammalian proteins were predicted using netnglyc-1.0. (c) Prediction of the position of the coronavirus 3C like protease cleaving protease in the protein using netcorona-1.0. (d) TMHMM (v2.0) was used to predict the transmembrane functional changes of S protein.

After sequence specificity and structure specificity of the S protein, four representative strains were randomly selected for protein function alignment according to region. Alignments to CH/LNFX/2022 and other lineages represent strain neutralizing epitopes via DNASTAR. A – V (aa 137), T-I (aa 559), and D – H (aa 1076) located at the NTD, S1/2, and HR-C showed differences from currently circulating PDCoV epitopes (Figure 6a). Interestingly, the remaining mutations, which did not alter the antigenic epitope, increased O-GalNAc glycosylation sites, implying that the highly glycosylated S protein can accelerate the glycosylation process and block the glycol-shield of antibody neutralizing viruses, equally unfavorable for vaccines and neutralizing antibodies to work (Figure 6b). Coronavirus 3C-like protease cleaves S protein as an important link in host invasion by coronaviruses. To explore whether mutations at the S1/2 junction of isolates affect this function, we predicted the cleavage site of S protein by Netcorona-1.0. A mutation of CH/LNFX/2022 at T-I (aa 559) that eliminates only one of the two cleavage sites was found by alignment of isolates and other prevalent strains (Figure 6c). In accordance with expectation, mutations located in the caudal HR-C domain did not alter the transmembrane function of the S protein (Figure 6d).

### Diarrhea in CH/LNFX/2022 newborn piglets

**Figure 7.**
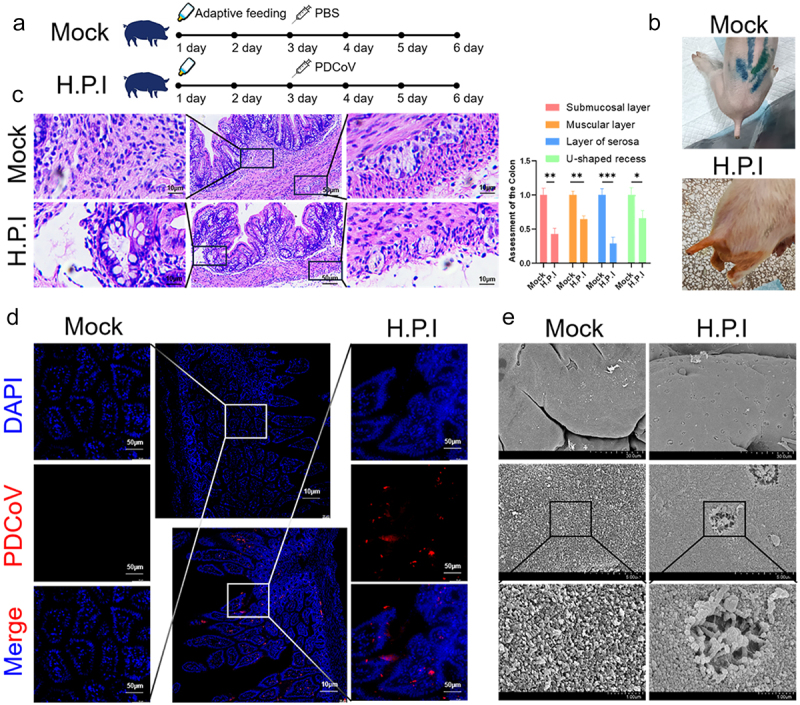
Pathological analysis of newborn piglets infected with highly pathogenic CH/LNFX/2022. (a) Newborn piglets were orally infected with PDCoV (*n* = 10) after 48 h artificial milk replacement, and the mock infection group was treated with PBS instead (*n* = 4). (b) Watery diarrhea occurred 24 h after infection and was normal in the mock infection group. (c) Colonic staining results were quantified using the mock infection group as a control (*n* = 3, *, *p* < 0.05; **, *p* < 0.01; ***, *p* < 0.001). (d) The animal model of infection was established by immunofluorescence of the jejunum (*n* = 3). (e) Results of scanning electron microscopy of the colon (*n* = 3).

To determine the histopathological changes in piglets infected with the PDCoV CH/LNFX/2022 strain, all piglets were adaptively fed with artificial milk substitute for 48 h, and the infected group was given PDCoV orally (TCID_50_ ≈ 10^6^), and the mock-infected group was given PBS orally, and tested 72 h after infection (Figure 7a). Overall results were similar in four piglets treated orally with CH/LNFX/2022, with watery feces at 48 h of infection, dehydration, and marked emaciation at 72 h, while normal in the simulated infection group (Figure 7b). The results of colon staining showed that compared with the mock infection group, the submucosa layer was significantly reduced (pink, **, *p* < 0.01), and fistulas were formed in some areas after CH/LNFX/2022 infection. In addition, muscular layer (orange, **, *p* < 0.01), serosal layer (blue, ***, *p* < 0.001), and U-shaped recess (green, *, *p* < 0.05) length were significantly reduced (Figure 7c). The immunofluorescence results of the jejunum of the piglets confirmed the establishment of the animal model. The infection group showed specific PDCoV fluorescence (red), while the mock infection group showed negative results (Figure 7d). The SEM results showed that the jejunal mucosa of newborn piglets infected with CH/LNFX/2022 developed a large area of ulceration, while the mock infection group was smooth and flat (Figure 7e), which corresponded to the results in Figure 7c.

## Discussion

Piglet diarrhea is a common epidemic disease in the swine industry, and the causes of piglet diarrhea are complex and diverse, but viral diarrhea can cause the most serious harm [24,25]. PDCoV is an emerging coronavirus, and swine infection is followed by symptoms such as vomiting, diarrhea, and lethality from dehydration [26]. In 2012, PDCoV was initially reported in Hong Kong, China, and in 2014, PDCoV became endemic from the United States and spread to several countries worldwide, including China, Japan, Korea, Thailand, and Canada, Mexico, and other countries and regions located in the Americas [27]. Vaccination is the most effective way to prevent and control pathogen transmission, but the commercialized PDCoV vaccine has not yet emerged and genomic diversity has caused great economic losses to the global swine industry [28,29].

So far, PDCoV has evolved into three lineages in China, America/Japan/Korea, and South-East Asia [30,31]. Since 2014, the Chinese lineage has been the main branch of PDCoV evolutionary transmission, and at the same time, it is of great significance to keep abreast of its epidemic trends and evolution as a reference lineage for existing vaccine development [32]. The S protein is the PDCoV major virulence associated glycoprotein and consists of a binding subunit S1 and a membrane fusion subunit S2, where S1 includes both NTD and CTD domains, has neutralizing epitopes that can stimulate the induction of neutralizing antibodies, and its RBD located at the CTD has a highly similar binding region to that on APNs of humans and pigs, which is the structural basis for PDCoV infection of humans and pigs [33]. At the same time, studies have shown that amino acid mutation and loss of S protein can affect the pathogenicity of the strain, and these results indicate that S protein plays an important role in influencing the virulence of coronavirus. In the present study, we show for the first time that S protein generates simultaneous 5-aa mutations at aa 137, aa 505, aa 559, aa 1076, and aa 1101, resulting in altered protein structure and function in the CH/LNFX/2022 NTD, S1/2, and HR-C regions (Figure 5 and 6). Meanwhile, we speculate that the A – V (aa 137), T-I (aa 559), and D – H (aa 1076) antigenic epitope alterations of CH/LNFX/2022 negatively impact the development of existing vaccines [34,35].

S2 includes the intrinsic membrane fusion peptide FP, peptide repeat HR, transmembrane adjacent region JMD, transmembrane domain TMD, and cytosolic region CD [36]. Upon binding of the RBD to the receptor, the S2 subunit changes conformation by insertion of the FP into the host cell membrane, with the HR arrangement bound into a 6HB that composes the fusion core leading to fusion of the virus with the cell membrane [37,38]. But it was discovered from the recent PDCoV epidemic that its glycosylation sites S-L (aa 505) and T-I (aa 559) for defense against vaccines and neutralizing antibodies have increased (Figure 6b). It is thought-provoking that three mutations in the 5-aa mutation alter the antigenic epitope and the remaining two increase a glycosylation site, which may be a unique evolutionary mechanism of immune evasion by PDCoV. Meanwhile, our study also found that T-I (aa 559) weakens a coronavirus 3C-like protease cleavage site, making the only two changed into one (Figure 6c). The gain of its glycosylation site and the disappearance of the coronavirus cleavage site may present difficulties for the in-depth study of PDCoV.

Adaptation of a strain to susceptible cells is an important link for strain isolation and identification. In this study, we detected the virus replication of CH/LNFX/2022 in ST cell culture P10 and P0 in tissue grinding solution (Figure 2e). The titer of P1 and P10 is not meaningful as P0 detection titer of tissue grinding solution. The ileum is more sensitive to enteric coronaviruses such as PDCoV and PEDV than the colon, and the lesions are mainly concentrated in the small intestine [39,40]. In this study, the immunofluorescence detection of PDCoV in the jejunum was used as the “lower limit” of the experiment to measure the success of the animal infection model. However, the morphological and ultrastructural observations of the colon, corresponding to the theme of the present study, on the basis of jejunal lesions were the validation of the highly pathogenic strain as the experimental “upper limit” (Supplementary Fig. S6). This is different from the previous general belief that there is no significant change in the colon after PDCoV infection [41–43]. This could be a unique change from the 5-aa mutation.

Genome recombination of different strains is an effective way to escape immune pressure, and cross-species recombination is particularly important. For example, the emergence of PDCoV HKU15 is the recombination between HKU17 and HKU11 in avian genus [44,45]. To further investigate the recombination relationship of CH/LNFX/2022, HUK11, HUK13, HUK16, HUK17, HUK19, HUK20, and HUK21 from different avian species were added as reference sequences, together with PDCoV reference sequences (Supplementary Table S3). The results show that CH/LNFX/2022 is a recombination of major parent CHN-HB-2014, CHN-SC2015, and minor parent Swine/Vietnam/Binh21/2015, HNZK-02. This indicates that the cross-species recombination of PDCoV may be nearing an end (Supplementary Fig. S7).

Coronaviruses have caused three major epidemics since 2003, including SARS-CoV-2, and in four genera of the family Coronaviridae, human infection is limited to alphacoronaviruses and betacoronaviruse [46]. In 2021, however, it was shown that PDCoV strains were identified in plasma samples from three Haiti children, the first human-related coronaviruses to infect outside the population, particularly in settings where close human to animal contact is possible [47]. Further studies revealed that the RBD of PDCoV is distinct from PEDV and has a highly similar binding region to human APN, providing a structural basis for infecting humans [48]. The latest study shows that the S protein of wild bird D-type coronavirus can invade cells of the corresponding species genus via chicken or swine APN mediated invasion, respectively, revealing the possibility of expanding the infected population by PDCoV [49]. Therefore, the detection of amino acid changes in the S protein can help to grasp its antigenic, pathogenic, and neutralizing epitope changes and has important public health implications for the development of vaccines and the establishment of assays [23].

## Conclusions

In this study, we analyzed the molecular characteristics and regression infection of CH/LNFX/2022 strain isolated from newborn piglets with diarrhea in China in 2022. The highly pathogenic CH/LNFX/2022 strain changed the three-domain epitope, added NTD and HR-C glycosylation sites, and reduced S1/2 coronavirus 3C-like protease cleavage sites. It also reduced the thickness of the small intestinal mucosa and disrupted the mucosal barrier. All the facts suggest that PDCoV is on a more complex path with important public health implications. Our results provide new insights into the establishment of high standard vaccines and diagnostics.

## Supplementary Material

Author Checklist E10 new.pdf

Supplementary Fig S6.tif

Supplementary Fig S5.tif

Supplementary Fig S3.tif

Supplementary Figure S legends.docx

Supplementary Fig S1.tif

Supplementary Fig S7.tif

Supplementary Fig S4.tif

Supplementary Fig S2.tif

Supplementary Table.doc

## Data Availability

The CH/LNFX/2022 and other DCoV sequences used in this study are available in GenBank of the National Center for Biotechnological Information. The accession numbers of all sequences are showed in Supplementary Table 1 and Table 3.
