# Supplementary figures and images for: The highly pathogenic strain of porcine deltacoronavirus disrupts the intestinal barrier and causes diarrhea in newborn piglets
Source: Virulence. 2025 Jan 6;16(1):2446742. doi: 10.1080/21505594.2024.2446742 (PMC12915422; doi:10.1080/21505594.2024.2446742)

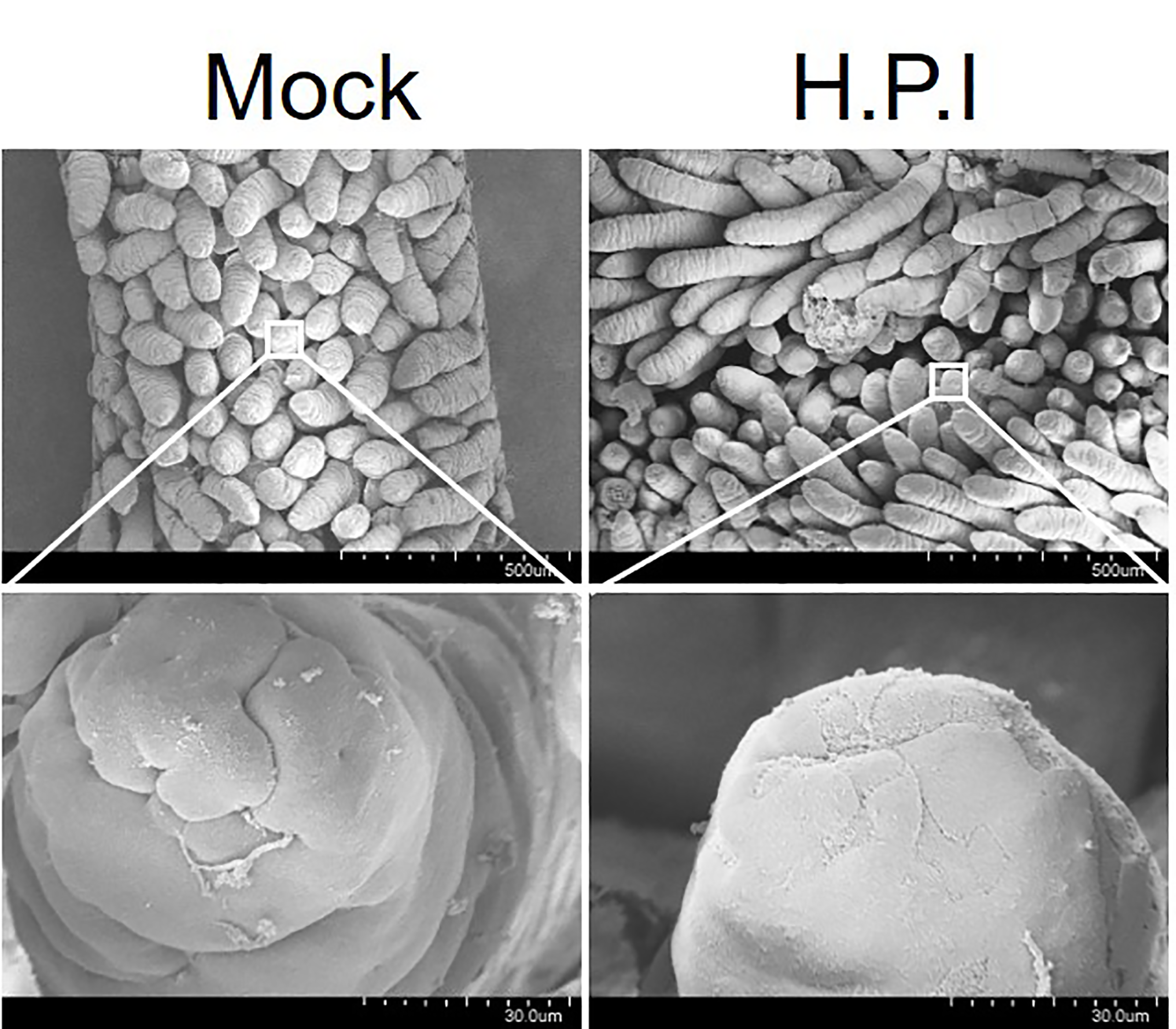

Supplement: Supplementary Fig S6.tif [file KVIR_A_2446742_SM3879.tif]

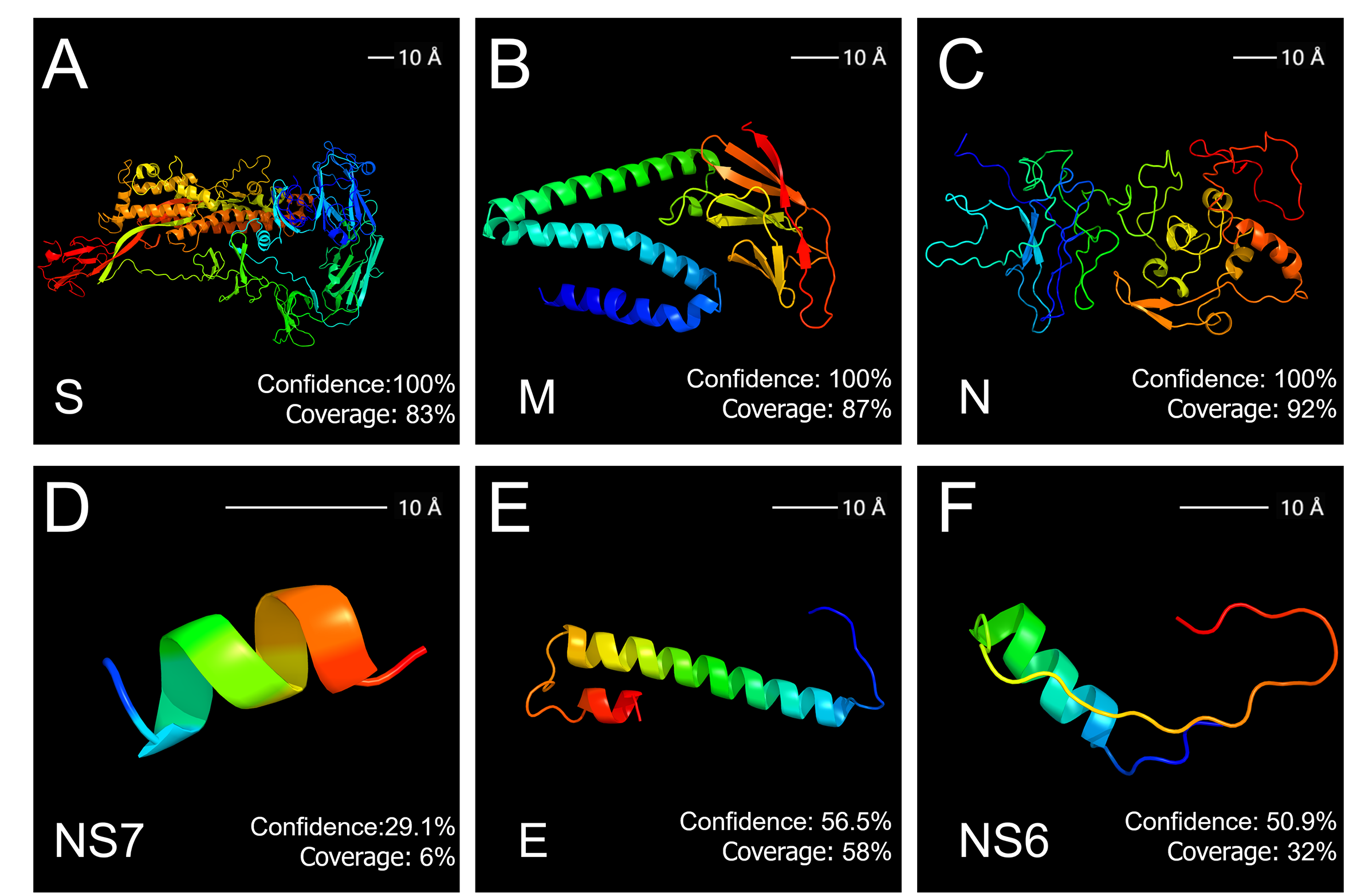

Supplement: Supplementary Fig S5.tif [file KVIR_A_2446742_SM3878.tif]

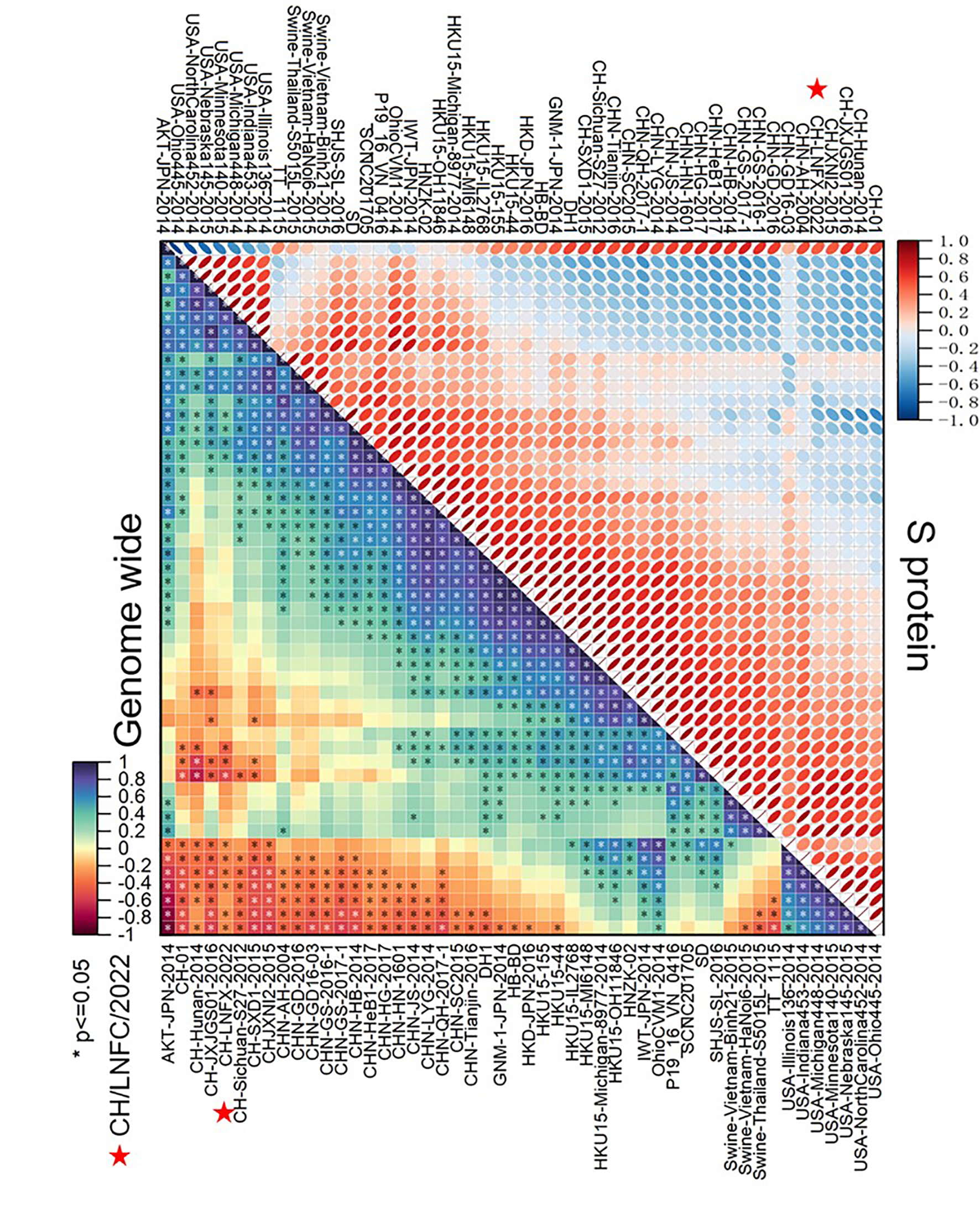

Supplement: Supplementary Fig S3.tif [file KVIR_A_2446742_SM3877.tif]

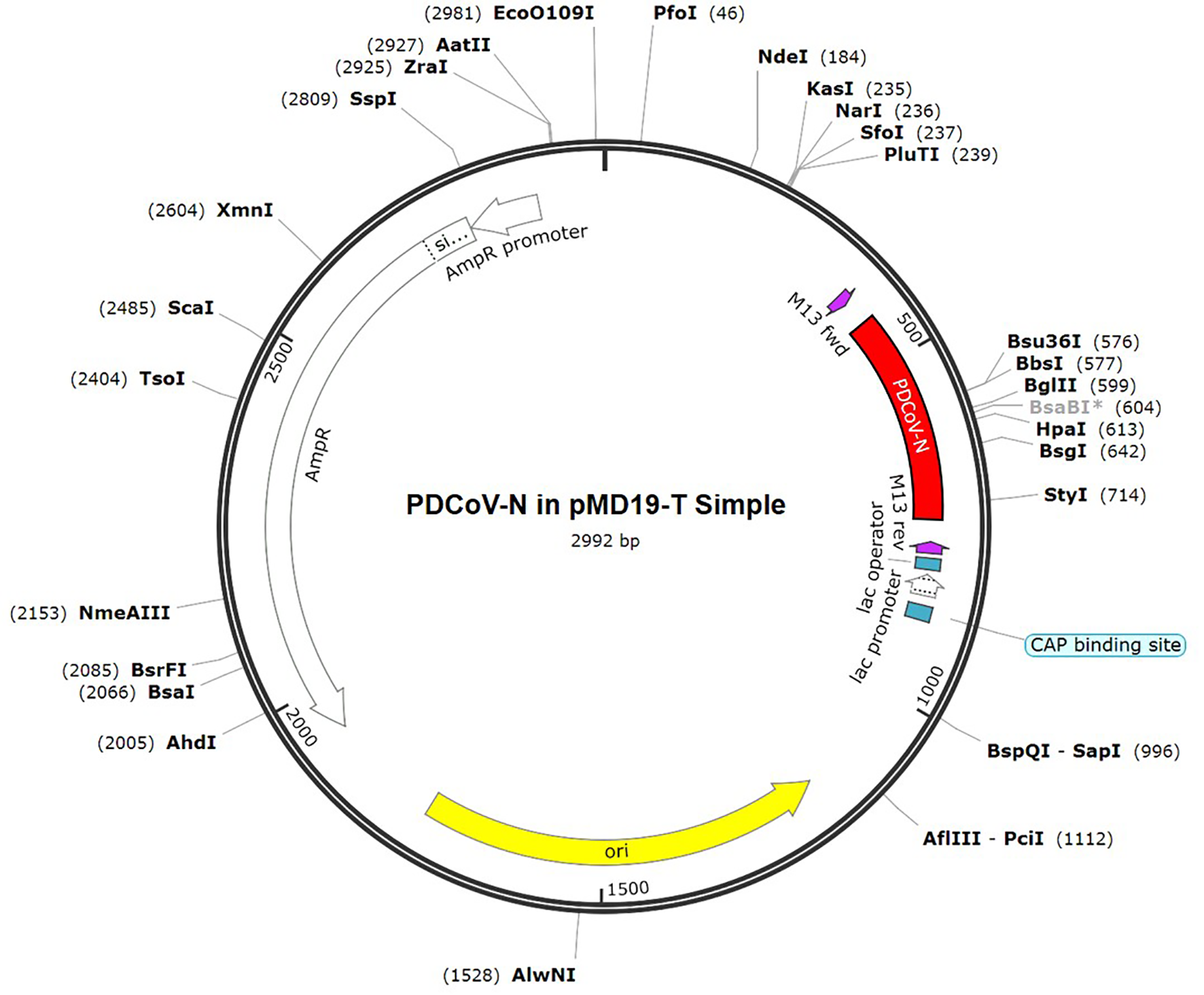

Supplement: Supplementary Fig S1.tif [file KVIR_A_2446742_SM3875.tif]

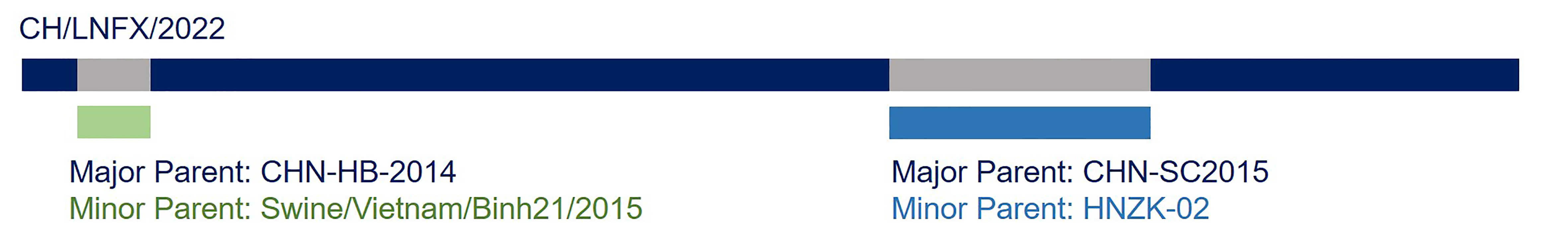

Supplement: Supplementary Fig S7.tif [file KVIR_A_2446742_SM3874.tif]

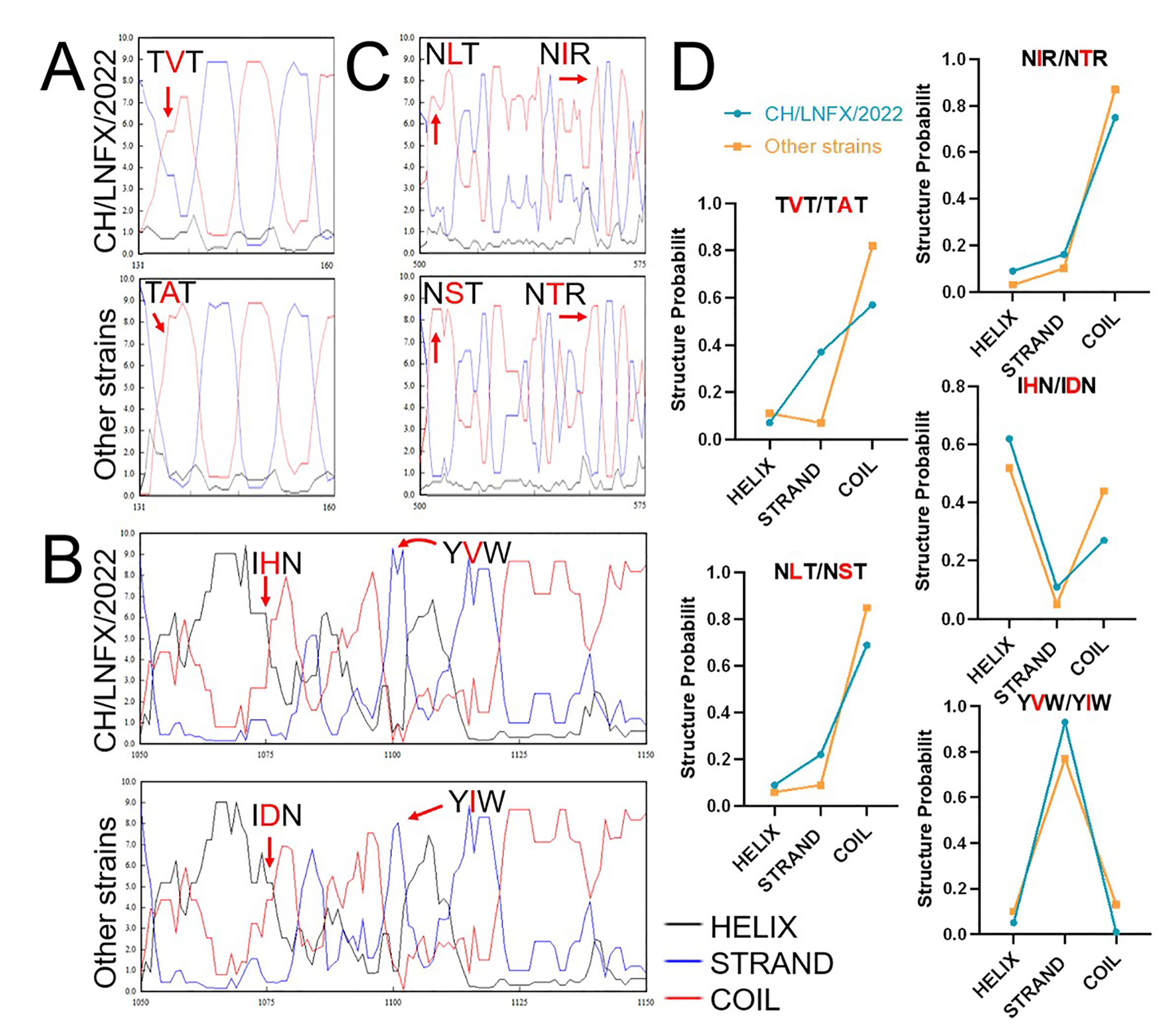

Supplement: Supplementary Fig S4.tif [file KVIR_A_2446742_SM3873.tif]

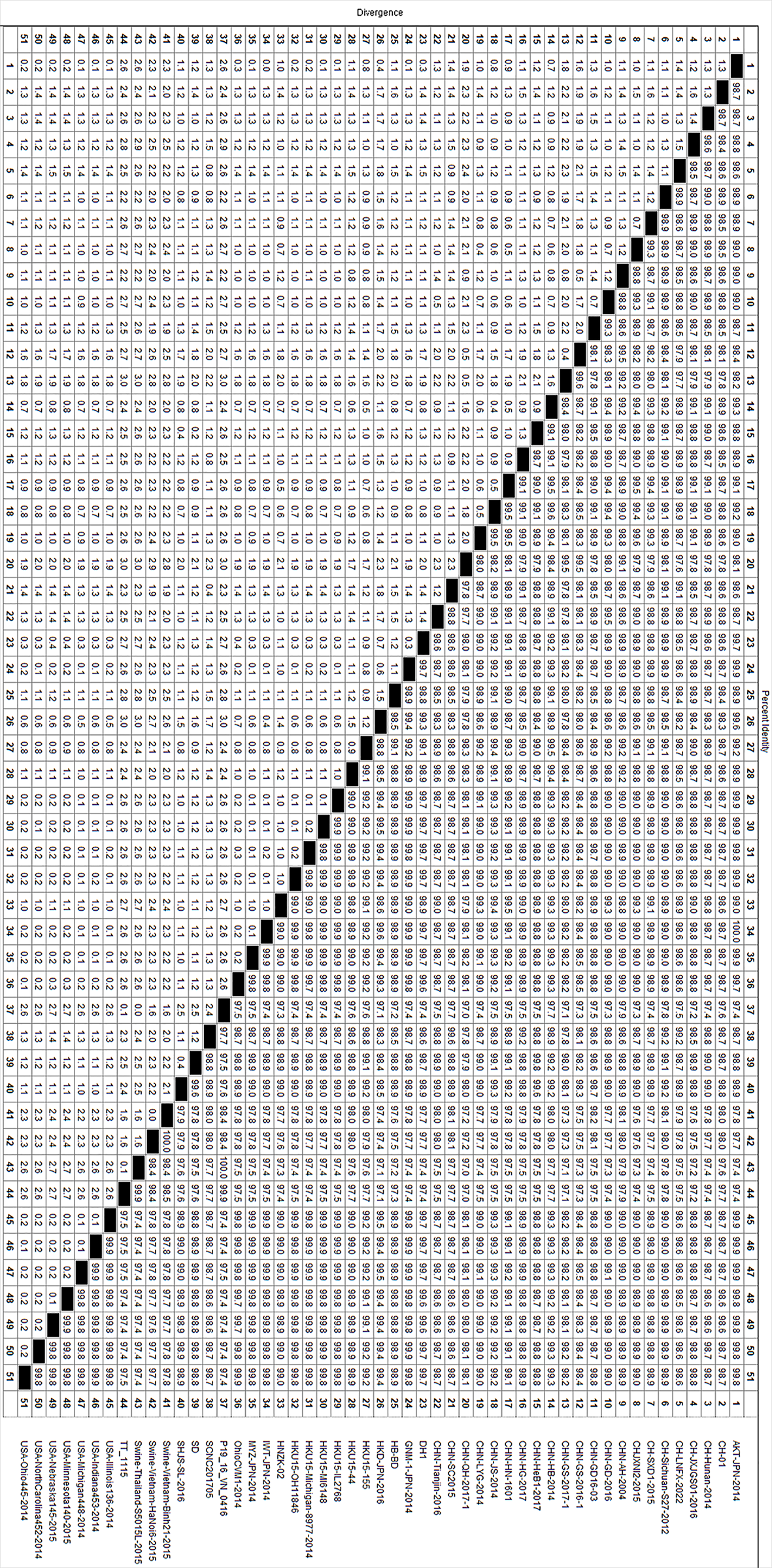

Supplement: Supplementary Fig S2.tif [file KVIR_A_2446742_SM3872.tif]
