# Supplementary material for: The highly pathogenic strain of porcine deltacoronavirus disrupts the intestinal barrier and causes diarrhea in newborn piglets
Source: Virulence. 2025 Jan 6;16(1):2446742. doi: 10.1080/21505594.2024.2446742 (PMC12915422; doi:10.1080/21505594.2024.2446742)
Supplement: Supplementary Figure S legends.docx [file KVIR_A_2446742_SM3876.docx]

***Supplementary Material***


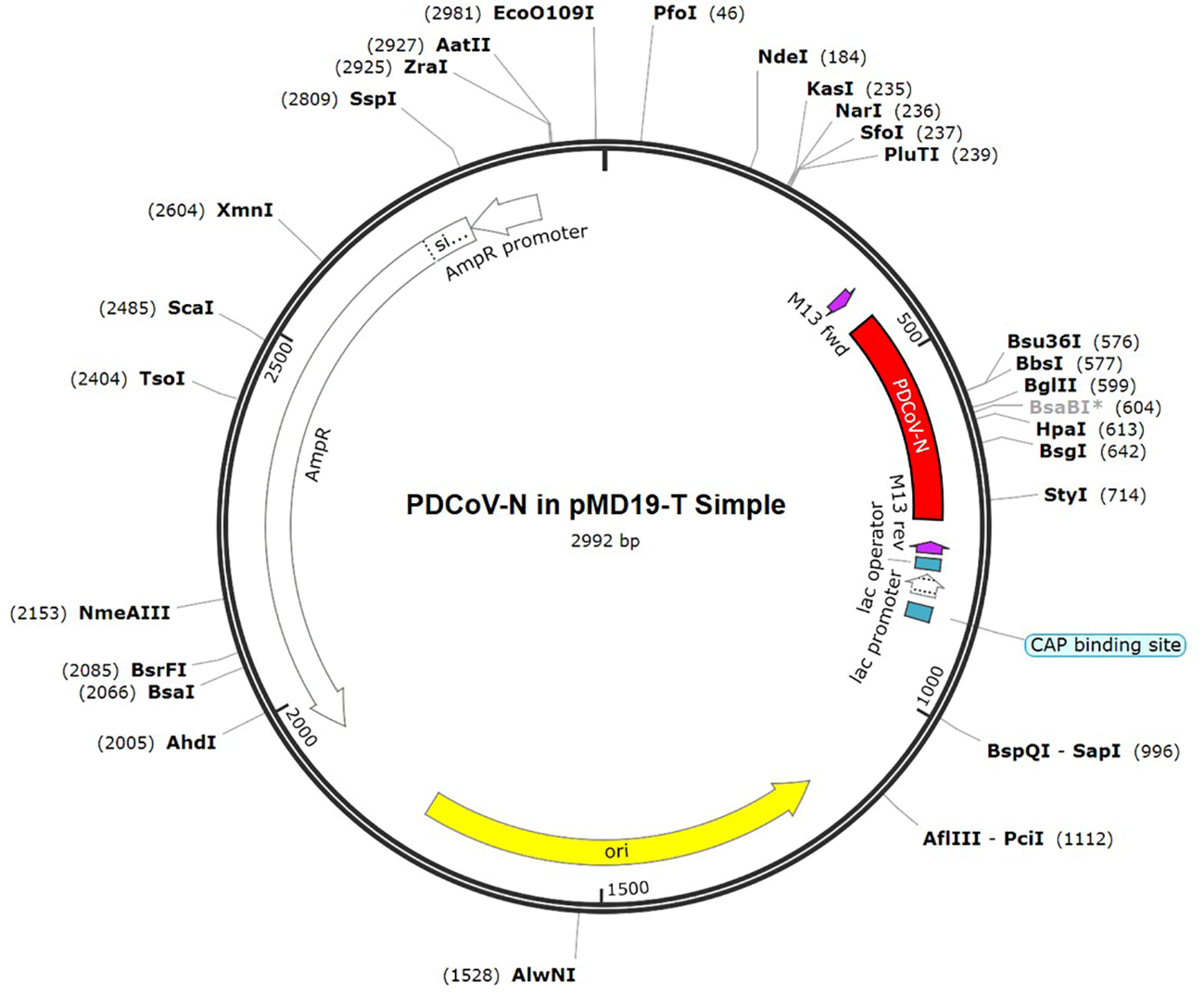


Supplementary Fig. 1. Schematic representation of the plasmid for cloning the partial sequence of the N gene of PDCoV.


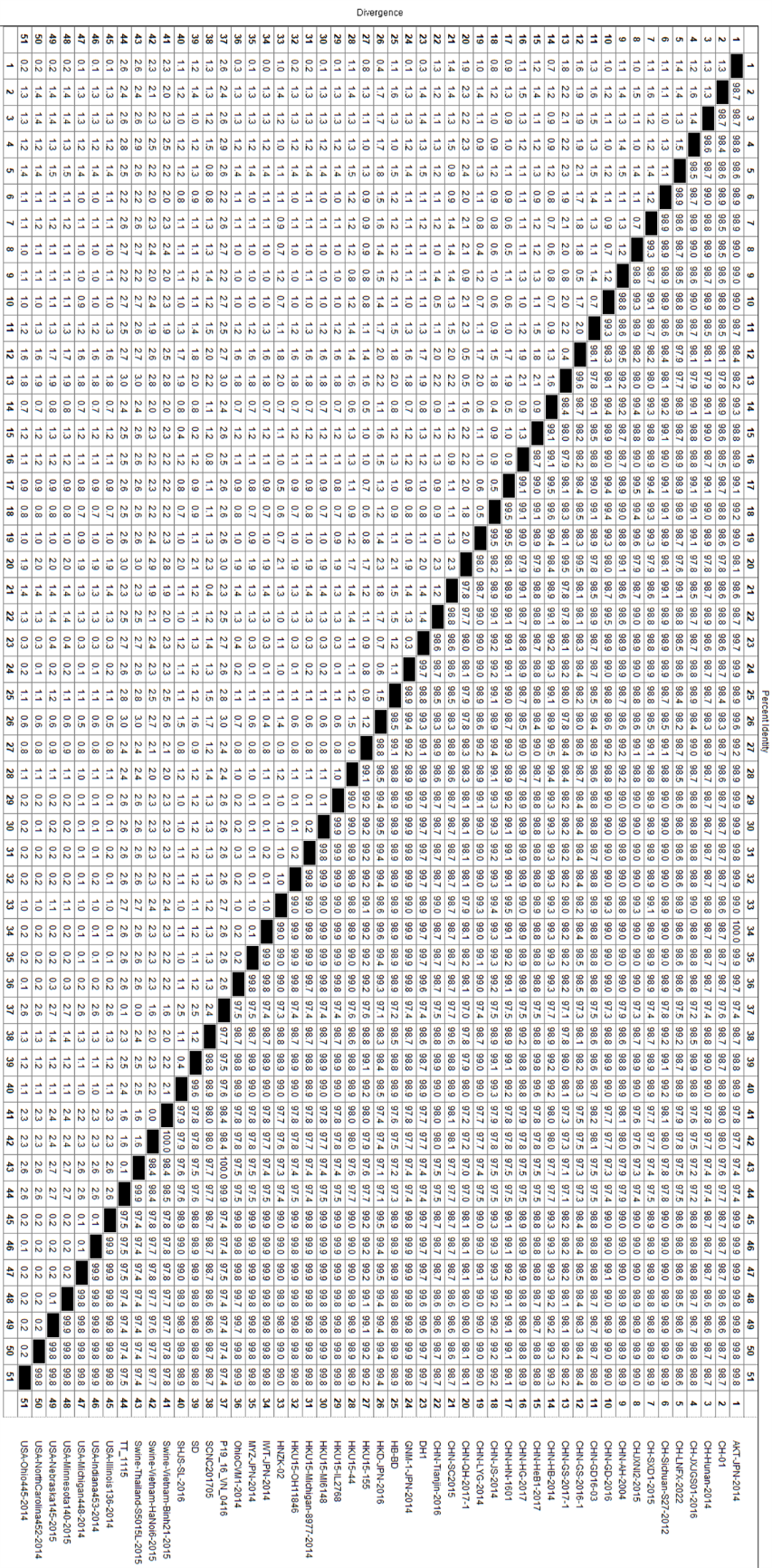


Supplementary Fig. 2. Genomic homology analysis of CH/LNFX/2022 with other 50 strains.


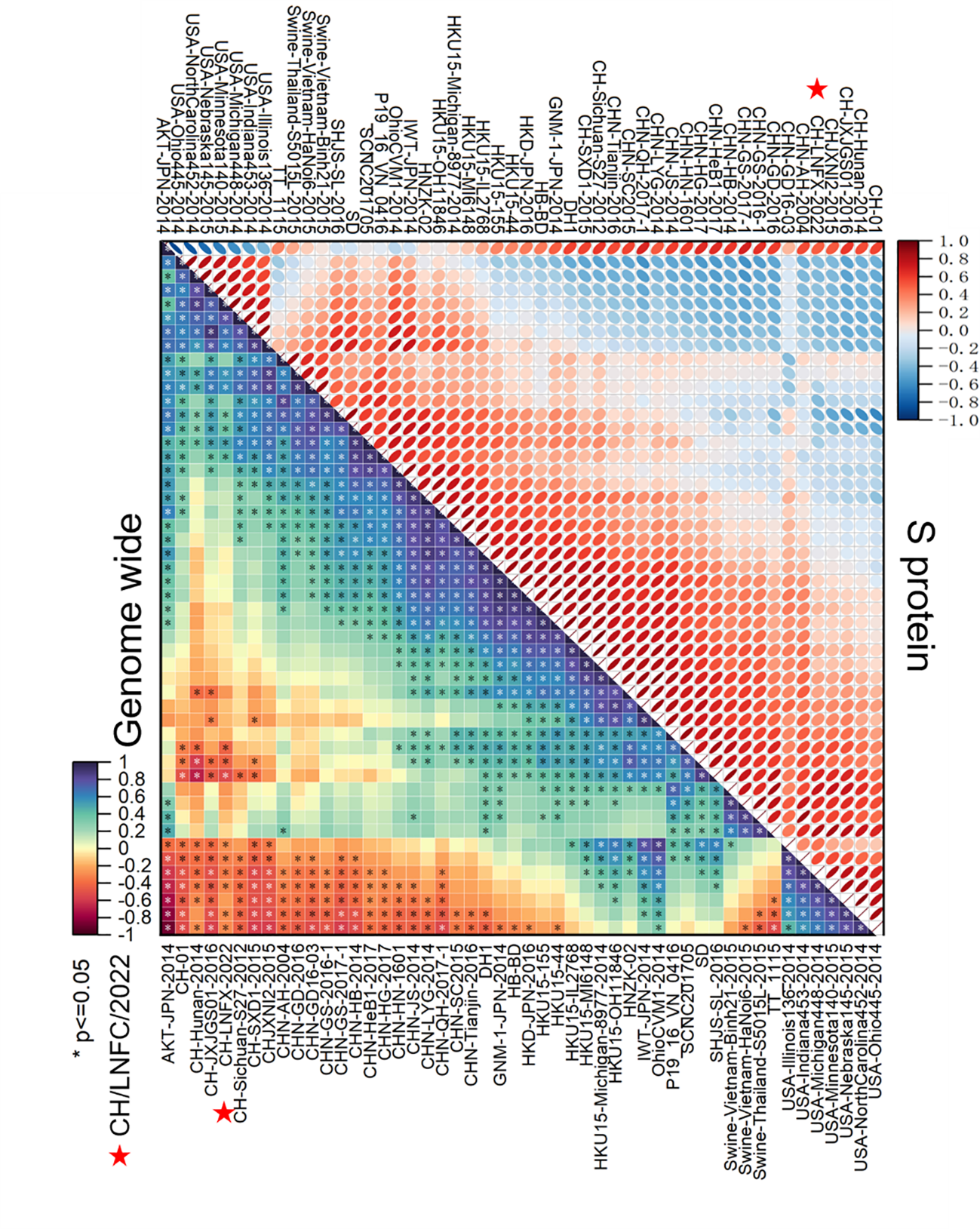


Supplementary Fig. 3. Analysis of the entire genome and homology of the S protein sequence of strain CH/LNFX/2022. The whole genomes of 51 viruses were analyzed for homology, and the homology results were subjected to a normalized Heatmap. The S protein was processed as above. * *P* ≤ 0.05 were considered statistically significant in the Heatmap.


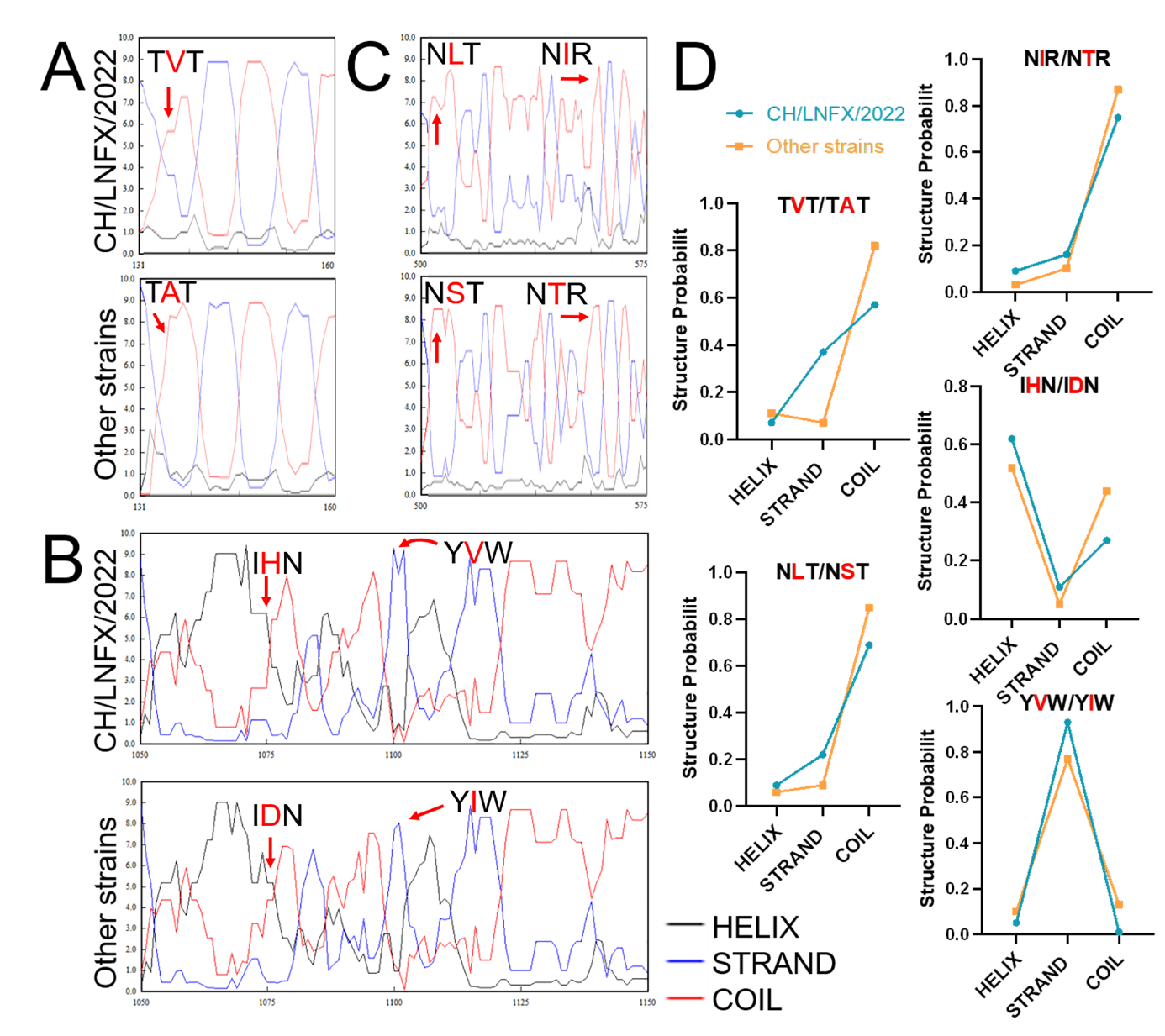


Supplementary Fig. 4. Prediction of S protein helix trends by DNAMAN. (A) A–V (aa 137) with the NTD structure. (B) D–H (aa 1076) and I–V (aa 1101) located in the HR-C domain. (C) S-L (aa 505) and T-I (aa 559). (D) NS6 protein to contrast. (E) The 5-aa mutation prediction is quantified, with light green representing CH/LNFX/2022 and light yellow representing other strains.


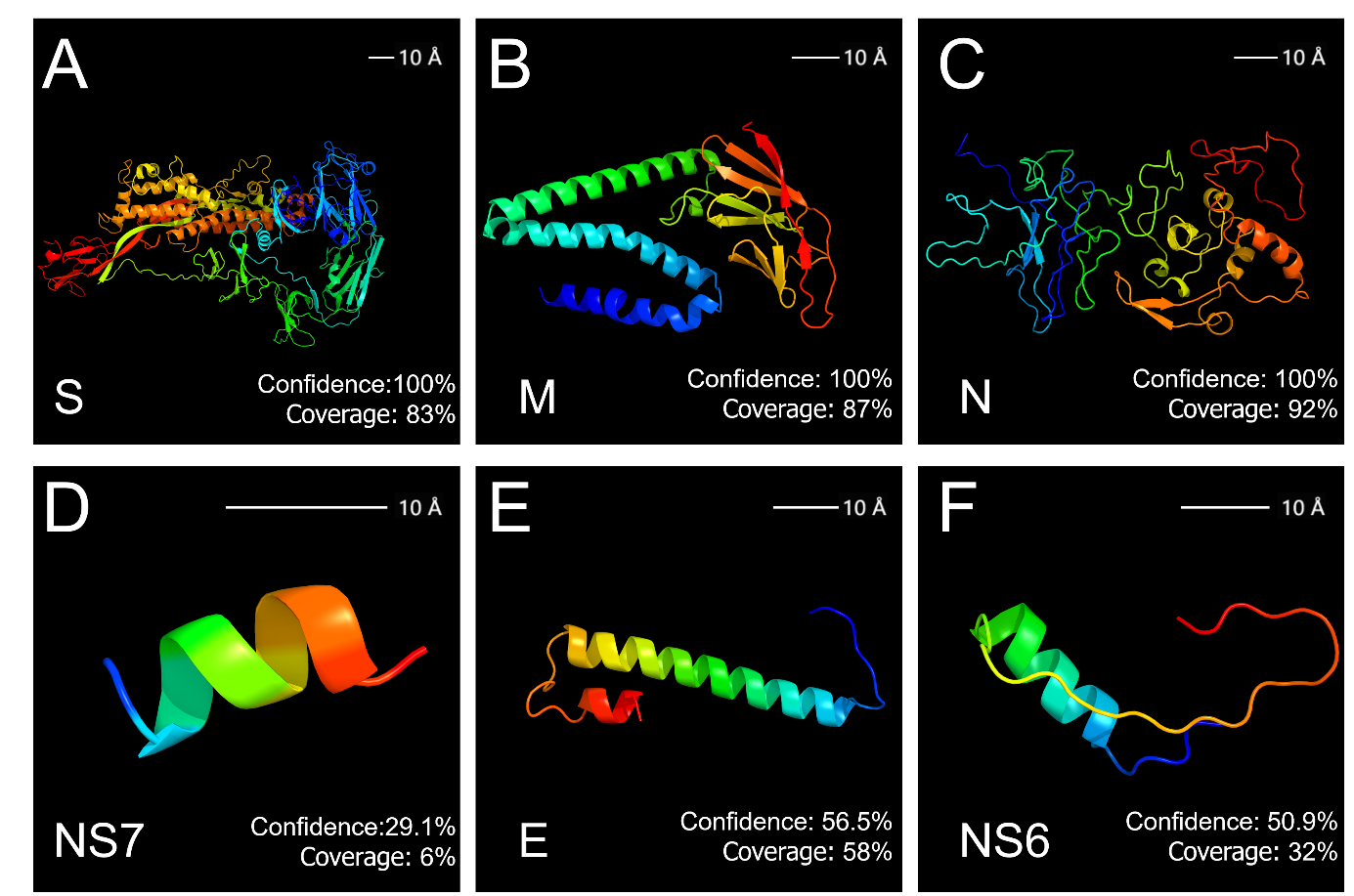


Supplementary Fig. 5. Protein tertiary structure modeling of CH/LNFX/2022 by Phyre2. From A to F are the S, M, N, NS7, E, and NS6 proteins in that order.


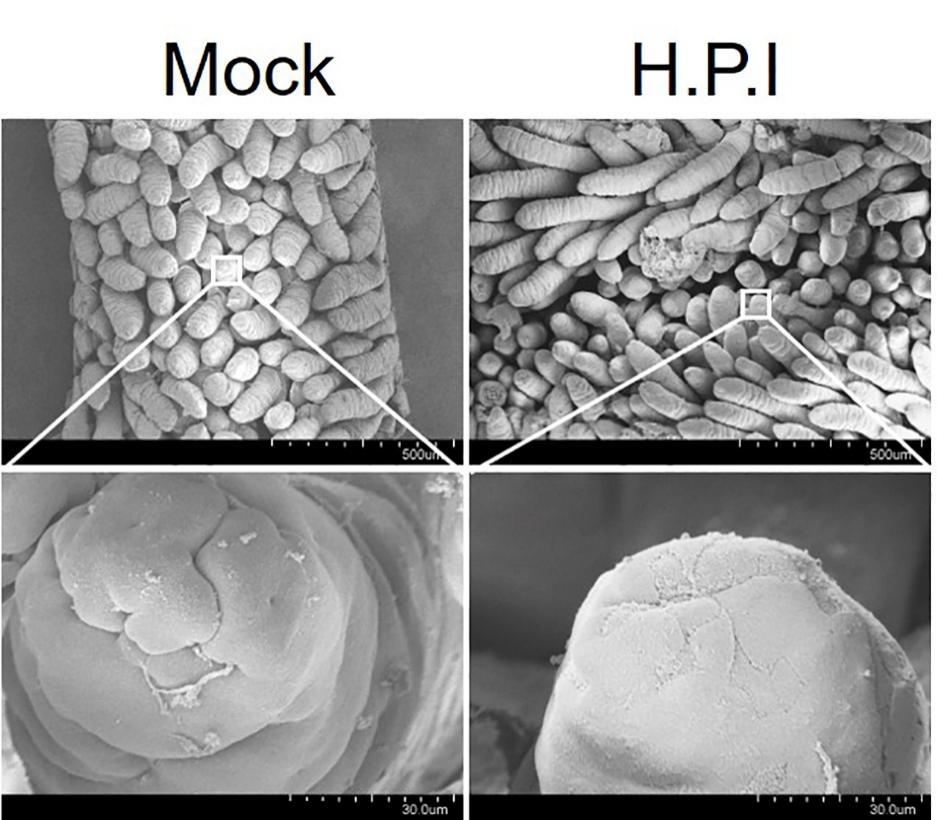


Supplementary Fig. 6. Scanning electron microscopy results of jejunum in mock infection group and infection group.


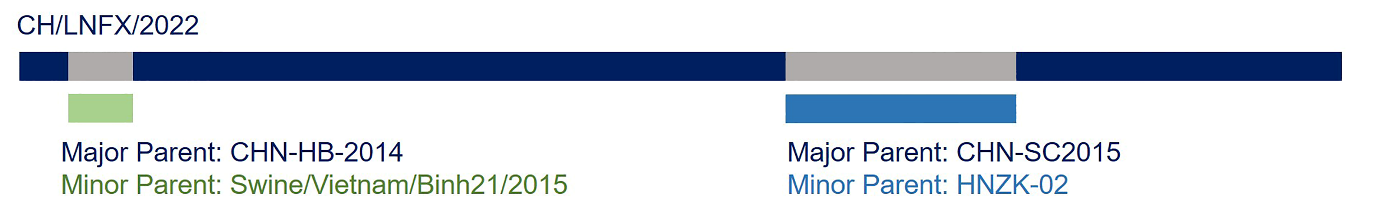


Supplementary Fig. 7. CH/LNFX/2022 was analyzed by recombination with PDCoV reference sequences and other DCoV reference sequences.
