# Supplementary material for: The highly pathogenic strain of porcine deltacoronavirus disrupts the intestinal barrier and causes diarrhea in newborn piglets
Source: Virulence. 2025 Jan 6;16(1):2446742. doi: 10.1080/21505594.2024.2446742 (PMC12915422; doi:10.1080/21505594.2024.2446742)
Supplement: Supplementary Table.doc [file KVIR_A_2446742_SM3871.doc]

Supplementary Table 1. PDCOV strains used in this study.

| Accession | Isolate | Collection Date | Geo Location |
| --- | --- | --- | --- |
| KX443143.2 | CH-01 | 2016 | China |
| KT266822.1 | CH/Sichuan/S27/2012 | 2012 | China |
| MF095123.1 | CHN-HG-2017 | 2017 | China |
| ON968724.1 | CH/LNFX/2022 | 2022 | China |
| MK572803.1 | SCNC201705 | 2017 | China |
| MK355396.1 | CHN-SC2015 | 2016 | China |
| KY513724.1 | CH/Hunan/2014 | 2014 | China |
| MF041982.1 | SHJS/SL/2016 | 2016 | China |
| MF431743.1 | SD | 2014 | China |
| MG242062.1 | CHN-HeB1-2017 | 2017 | China |
| KP757892.1 | CHN-JS-2014 | 2014 | China |
| KY363867.1 | CHN-GD16-03 | 2016 | China |
| KY065120.1 | CHN/Tianjin/2016 | 2016 | China |
| MF280390.1 | CHN-GD-2016 | 2016 | China |
| MH708123.1 | HNZK-02 | 2018 | China |
| MG832584.1 | CHN-HN-1601 | 2016 | China |
| KT021234.1 | CH/SXD1/2015 | 2015 | China |
| KU665558.1 | CHN-LYG-2014 | 2014 | China |
| KR131621.1 | CHJXNI2/2015 | 2015 | China |
| MF948005.1 | HB-BD | 2017 | China |
| MK625641.1 | CH/JXJGS01/2016 | 2018 | China |
| NC039208.1 | HKU15-155 | 2010 | China |
| KP757891.1 | CHN-HB-2014 | 2014 | China |
| JQ065042.2 | HKU15-44 | 2009 | China |
| KP757890.1 | CHN-AH-2004 | 2004 | China |
| MF642322.1 | CHN/GS/2016/1 | 2016 | China |
| MF642325.1 | CHN/QH/2017/1 | 2017 | China |
| MF642324.1 | CHN/GS/2017/1 | 2017 | China |
| KR265863.1 | USA/Ohio445/2014 | 2014 | USA |
| KJ769231.1 | OhioCVM1/2014 | 2014 | USA |
| KR265850.1 | USA/Michigan448/2014 | 2014 | USA |
| KM012168.1 | Michigan/8977/2014 | 2014 | USA |
| KJ584355.1 | HKU15 strain IL2768 | 2014 | USA |
| KR265851.1 | USA/Indiana453/2014 | 2014 | USA |
| KX022605.1 | USA/Nebraska145/2015 | 2015 | USA |
| KX022603.1 | USA/Minnesota140/2015 | 2015 | USA |
| KT381613.1 | HKU15-OH11846 | 2014 | USA |
| KJ620016.1 | HKU15-MI6148 | 2014 | USA |
| KR265858.1 | USA/NorthCarolina452/2014 | 2014 | USA |
| KJ601779.1 | USA/Illinois136/2014 | 2014 | USA |
| KY354363.1 | DH1 | 2016 | South Korea |
| LC260039.1 | GNM-1/JPN/2014 | 2014 | Japan |
| LC260045.1 | HKD/JPN/2016 | 2016 | Japan |
| LC260041.1 | IWT/JPN/2014 | 2014 | Japan |
| LC260038.1 | AKT/JPN/2014 | 2014 | Japan |
| KX834351.1 | Vietnam/HaNoi6/2015 | 2015 | Viet Nam |
| KX834352.1 | Vietnam/Binh21/2015 | 2015 | Viet Nam |
| MH118332.1 | P19_16_VN_0416 | 2016 | Viet Nam |
| KU984334.1 | TT_1115 | 2015 | Thailand |
| KU051649.1 | Thailand/S5015L/2015 | 2015 | Thailand |

Supplementary Table 2. The primer sequences.

| Type of Virus | Name of Primer | Sequence of 5’-3’ | Target gene | Fragment length |
| --- | --- | --- | --- | --- |
| PEDV | ORF3-F | ATGTTTCTTGGACTTTTTC | ORF3 | 675bp |
|  | ORF3-R | TCATTCACTAATTGTAGCATAC |  |  |
| PCV2 | ORF2-F | CGGATATTGTAGTCCTGGTCG | ORF2 | 481bp |
|  | ORF2-R | ACTGTCAAGGCTACCACAGTC |  |  |
| PDCoV | N-F | ATGGCTACTGGCTGCGTTAC | N | 383bp |
|  | N-R | GCGTTTCCTGGGCTGATT |  |  |
|  | N-qF | AGCTCCCAAGCGGACTTTACCCAA |  | 112bp |
|  | N-qR | AGCCATACCCGTCTTCTCAGTGTC |  |  |
| TGEV | S2-F | GTGGTTTTGGTYRTAAATGC | S | 859bp |
|  | S2-R | CACTAACCAACGTGGARCTA |  |  |
| PRRSV | Nsp2-F | ATGTTGTGCTTCCTGGGGTTG | Nsp2 | 600-1kbp |
|  | Nsp2-R | CTTGACAGGGAGCTGCTTGA |  |  |
| PRV | gD-F | GGTGGACCGGCTGCTGAACGA | gD | 455bp |
|  | gD-R | GCTGCTGGTAGAACGGCGTCA |  |  |
| BVDV | 5’UTR-F | GGTAGCAACAGTGGTGAG | 5’UTR | 220bp |
|  | 5’UTR-R | GTAGCAATACAGTGGGCC |  |  |

Supplementary Table 3. Other DCoV strains were used in this study.

| Accession | Isolate | Collection Date | Geo Location |
| --- | --- | --- | --- |
| FJ376620.1 | HKU11-796 | 2007 | China |
| NC_011550.1 | HKU13-3514 | 2007 | China |
| NC_016991.1 | HKU16 | 2007 | China |
| NC_016992.1 | HKU17 | 2007 | China |
| NC_016994.1 | HKU19 | 2007 | China |
| NC_016995.1 | HKU20 | 2008 | China |
| NC_016996.1 | HKU21 | 2007 | China |
